# Gut microbial metabolite butyrate boosts p53-expressing telomerase-specific oncolytic adenovirus efficacy by enhancing infectivity and activating MHC-I/cGAS-STING

**DOI:** 10.1007/s00262-025-04252-4

**Published:** 2025-12-18

**Authors:** Masaki Sakamoto, Shinji Kuroda, Tetsuya Katayama, Yu Mikane, Shunya Hanzawa, Daisuke Kadowaki, Yusuke Yoshida, Yuki Hamada, Ryoma Sugimoto, Chiaki Yagi, Masashi Hashimoto, Nobuhiko Kanaya, Yoshihiko Kakiuchi, Satoru Kikuchi, Kunitoshi Shigeyasu, Hiroshi Tazawa, Shunsuke Kagawa, Yasuo Urata, Toshiyoshi Fujiwara

**Affiliations:** 1 Department of Gastroenterological Surgery, Okayama University Graduate School of Medicine, Dentistry and Pharmaceutical Sciences, 2-5-1 Shikata-cho, Kita-ku, Okayama, 700-8558 Japan; 2 Center for Innovative Clinical Medicine, Okayama University Hospital, Okayama, Japan; 3 Oncolys BioPharma, Inc., Tokyo, Japan

**Keywords:** Butyrate, Oncolytic adenovirus, MHC-I, CD8 + T cells, Cancer immunotherapy

## Abstract

The gut microbiota plays an essential role in regulating host immunity, and its metabolites such as butyrate exert immunomodulatory effects by acting as histone deacetylase inhibitors. Oncolytic virotherapy has emerged as a promising approach for cancer treatment, and we have developed OBP-702, a telomerase-specific oncolytic adenovirus that expresses p53 and elicits strong systemic antitumor responses. In this study, the potential synergy between butyrate and OBP-702 was investigated in colorectal cancer models. Using human and murine colorectal carcinoma cell lines, butyrate was found to directly enhance the infectivity of OBP-702 by upregulating CAR and integrins, thereby promoting apoptosis and autophagy in tumor cells. In addition, butyrate indirectly boosted systemic antitumor immunity by upregulating MHC-I expression through activation of the cGAS-STING pathway and enhancing CD8 + T cell recruitment via CXCL10 secretion. These findings were supported by in vivo experiments using CT26 subcutaneous, bilateral, and orthotopic tumor models, in which the combination of oral butyrate and intratumoral OBP-702 administration produced synergistic antitumor effects. These results highlight the therapeutic potential of integrating gut microbial metabolites with oncolytic virotherapy as a novel immunotherapeutic strategy for colorectal cancer.

**Supplementary Information:**

The online version contains supplementary material available at 10.1007/s00262-025-04252-4.

## Introduction

The term “gut microbiota” refers to the diverse community of microorganisms that live in the digestive tract and play a crucial role in maintaining health. The majority of these microorganisms reside in the colon and contribute to various functions, including digestion, metabolism, and immune regulation. The composition of gut microbiota can be affected by factors such as diet, genetics, age, and medication. Imbalances in gut microbiota, known as dysbiosis, have been associated with a range of diseases, including inflammatory bowel disease, diabetes mellitus, and cancer [1, 2]. Colorectal cancer (CRC) is the third most common cancer in terms of incidence and the second leading cause of cancer-related mortality worldwide [3], and its development has been reported to be associated with changes or imbalances in gut microbiota. Although the specific bacterial species remain unclear, recent studies suggest that certain metabolites produced by gut microbiota, such as short-chain fatty acids (SCFAs), can influence inflammation and immune responses in the colon, potentially affecting the progression of CRC [4, 5].

Butyrate, one of the SCFAs, is produced in the colon through bacterial fermentation of dietary fibers and starch [6], and it has several important roles in maintaining gut health and preventing cancer development, such as anti-inflammatory effects, induction of apoptosis, regulation of cell differentiation and proliferation, and maintenance of epithelial barrier integrity [7]. Butyrate, a histone deacetylase (HDAC) inhibitor, upregulates the expression of major histocompatibility complex (MHC) class I (MHC-I), which is a molecule that presents intracellular peptides such as tumor antigen to CD8 + cytotoxic T cells, and it plays a crucial role in the antitumor immune response [8]. The expression of MHC-I is regulated through the cGAS-STING pathway, which is affected by HDAC inhibition, with butyrate playing a role in this regulation as an HDAC inhibitor [9, 10]. Thus, butyrate is expected to enhance the antitumor immune response and produce synergistic effects with immune checkpoint inhibitors.

Oncolytic virotherapy is an innovative approach to cancer treatment that leverages the ability of viruses to selectively infect and kill tumor cells while stimulating a robust immune response [11]. While several oncolytic viruses, such as Talimogene laherparepvec (T-VEC) and Teserpaturev, have been approved for cancer treatment and are currently in clinical use [12, 13], we have also developed telomerase-specific oncolytic adenoviruses. One of them is the first-generation virus agent, OBP-301 (Suratadenoturev), which is currently being tested in a phase 2 clinical trial for esophageal cancer in combination with radiotherapy [14–17]. We have developed the second-generation viral agent, OBP-702, which incorporates the p53 tumor suppressor gene into the basic structure of OBP-301, and we have demonstrated that OBP-702 exhibited more profound antitumor activity than OBP-301 [18, 19]. Interestingly, OBP-702 has the potential to strongly activate antitumor immunity even in pancreatic cancer, a representative cold tumor, by inducing immunogenic cell death, activating dendritic cells, facilitating the recruitment of CD8 + T cells, activating memory T cells, and suppressing myeloid-derived suppressor cells and cancer-associated fibroblasts [20–23].

In the present study, the aim was to evaluate the combined effect of the gut microbial metabolite butyrate and the oncolytic adenovirus OBP-702 on CRC, focusing on the effect of butyrate on the antitumor activity of OBP-702. The underlying mechanisms of this effect were also elucidated, considering both the direct effects of butyrate on OBP-702 and the indirect effects mediated through the immune response. By investigating the role of gut microbiota, particularly the therapeutic potential of butyrate, this study could provide insights that may transform CRC treatment and lay the foundation for future clinical trials combining gut microbiota manipulation with oncolytic virus therapy.

## Materials and methods

### Cell lines and culture conditions

The murine colon carcinoma cell lines (CT26, MC38), the human colon carcinoma cell lines (SW48, HCT116, RKO), the murine macrophage cell line (RAW264.7), and the human monocytic leukemia cell line (THP-1) were purchased from the American Type Culture Collection (ATCC, Manassas, VA, USA). CT26, SW48, RAW264.7, and THP-1 cells were cultured in RPMI 1640 medium; MC38 cells were cultured in Dulbecco’s Modified Eagle Medium (DMEM); HCT116 cells were cultured in McCoy’s 5A medium; and RKO cells were cultured in Eagle’s Minimum Essential Medium (EMEM). All media were supplemented with 10% fetal bovine serum (FBS) and 1% penicillin–streptomycin (100 U/mL). THP-1 cells were differentiated into macrophage-like cells by treatment with 100 ng/mL phorbol 12-myristate 13-acetate (PMA) for 24 h, followed by a 48 h rest period in fresh medium before experiments. After PMA treatment, the cells adhered to the culture dish, indicating successful differentiation. All cell lines were maintained at 37 °C in a humidified atmosphere containing 5% CO_2_. None of the cell lines was cultured for more than 3 months after thawing, and cell authentication was not performed by the authors.

### Adenoviruses

OBP-702 is a p53-expressing telomerase-specific oncolytic adenovirus, in which the human telomerase reverse transcriptase (hTERT) promoter drives the expression of the E1A and E1B genes, and the early growth response 1 (Egr1) promoter drives the expression of the p53 gene (Fig. S1). Ad-GFP is a replication-deficient adenovirus expressing green fluorescent protein (GFP). Multiplicity of infection (MOI) and plaque-forming units (PFUs) were used as virus units in vitro and in vivo, respectively.

### Butyrate

Butyrate was purchased from Sigma-Aldrich (B103500, St. Louis, MO, USA). For in vitro experiments, butyrate was prepared in culture medium at concentrations of 0, 1, 2, and 3 mM and added to the cell cultures. For in vivo studies, butyrate was diluted in drinking water to a final concentration of 1 mM, and mice were provided with the butyrate-containing water daily throughout the experimental period.

Butyrate concentration was measured with the following protocol. To each sample, 250 μL of methanol were added, and the samples were deproteinized with methanol. After vortexing for 30 s, the samples were centrifuged at 16,000 g for 3 min at 25 °C. A total of 20 μL of methanol containing an internal standard were added to 180 μL of the supernatant from each sample. The supernatants were analyzed using the Shimadzu single quadrupole GCMS-QP2020 NX gas chromatograph-mass spectrometer (GC–MS) (Kyoto, Japan).

### Cell viability assay

CT26, MC38, SW48, HCT116, and RKO cells were seeded in 96-well plates at a density of 1 × 10^3^ cells/well. The cells were treated with butyrate, followed by OBP-702 treatment at the indicated doses 2 h later. Cell viability was assessed 3 days after OBP-702 treatment using a Cell Proliferation Kit II (XTT) (Roche Diagnostics, Basel, Switzerland) according to the manufacturer’s protocol. The percentage of viable cells relative to untreated control cells was calculated, and all experiments were performed in triplicate.

To evaluate the efficacy of the combination therapy of OBP-702 and butyrate, the combination index (CI) was calculated using CalcuSyn software (BIOSOFT, Acropolis Computers Ltd., Cambridge, UK). The CI is a quantitative measure that indicates the extent of synergism (CI < 1), additive effect (CI = 1), or antagonism (CI > 1) between two agents. The CI was calculated based on the median-effect principle using the following formula: CI = (D)1/(Dx)1 + (D)2/(Dx)2, where (D)1 and (D)2 are the doses of agent 1 and agent 2 used in combination to achieve an x% effect, and (Dx)1 and (Dx)2 are the doses of agent 1 and agent 2 that would achieve the same effect when used alone.

### Western blot analysis

Proteins extracted from whole-cell lysates were electrophoresed on 10–15% SDS–polyacrylamide gels and transferred onto Hybond-polyvinylidene difluoride (PVDF) membranes (GE Healthcare UK, Amersham, UK). The membranes were incubated overnight at 4 °C with primary antibodies against p53 (1:1,000, cat. 18,032, Cell Signaling Technology, Danvers, MA, USA), E1A (1:1,000, cat. 554,155, BD Pharmingen, San Jose, CA, USA), p62 (1:1,000, cat. 5114, Cell Signaling Technology), PARP (1:1,000, cat. 9542, Cell Signaling Technology), CAR (1:1,000, cat. 18,979, Santa Cruz Biotechnology, Dallas, TX, USA), and β-actin (1:5,000, cat. A-5441, Sigma-Aldrich). After washing, the membranes were incubated with peroxidase-conjugated secondary antibodies for 1 h at room temperature. The Amersham enhanced chemiluminescence (ECL) system (GE Healthcare UK) was used to detect the peroxidase activity of the bound antibodies. Equal protein loading was confirmed by probing for β-actin.

### Flow cytometry

Flow cytometry was performed for CAR (D3W3G, Cell Signaling Technology), αvβ3 (bs-1310R, Bioss antibodies, Woburn, MA, USA), αvβ5 (bs-1356R, Bioss Antibodies), and MHC-I (BE0077, Bio X Cell, Lebanon, NH, USA) using FACS Array and BD FACS Aria (BD Biosciences, San Jose, CA, USA), and analysis was performed using FlowJo software (BD Biosciences).

For the in vivo experiments, cells were incubated with Zombie Aqua Fixable Viability Kit (BioLegend, San Diego, CA, USA) and Fc Block (BioLegend) for 20 min on ice. Single cell suspensions were stained for surface markers (MHC-I [BE0077, Bio X Cell], NLRC5 [DF13672, Affinity Bioscience], CD45 [30-F11, BioLegend], CD8a [YTS169.4, Abcam, Cambridge, UK] and intracellular cytokines (IFN-γ [XMG1.2, BioLegend], Granzyme B [QA18A28, BioLegend]) in PBS for 15 min at room temperature. For intracellular staining of IFN-γ and Granzyme B, cells were treated with GolgiStop (BD Biosciences) for 4 h prior to staining.

### Immunostaining

Cells incubated for 24 h after Ad-GFP infection were washed with PBS and counterstained with DAPI to visualize the nuclei. GFP expression was observed using an inverted fluorescence microscope equipped with a GFP filter. Images were captured using standardized exposure settings to ensure comparability.

In experiments using freshly frozen samples of subcutaneous tumors harvested 2 days after Ad-GFP infection, cryosections (4 μm) were observed using an inverted fluorescence microscope equipped with a GFP filter.

For immunohistochemistry, formalin-fixed, paraffin-embedded tissue samples (4 μm) were deparaffinized in xylene and rehydrated using a graded ethanol series. After blocking endogenous peroxidases by incubation with 3% H_2_O_2_ for 10 min, the samples were boiled in citrate buffer or EDTA buffer for 14 min in a microwave oven for antigen retrieval. The samples were incubated with primary antibodies against CD8 (eBioscience, San Diego, CA, USA) for 1 h at room temperature or overnight at 4 °C and then with peroxidase-conjugated secondary antibody for 30 min at room temperature. Samples were stained with 3,3-diaminobenzidine for signal detection, counterstained with Mayer’s hematoxylin, and then dehydrated and mounted onto coverslips.

For fluorescent immunohistochemistry, formalin-fixed, paraffin-embedded tumor Sects. (4 μm) were deparaffinized in xylene, rehydrated through a graded ethanol series, and subjected to antigen retrieval using citrate buffer. Nonspecific binding was blocked with a blocking solution for 1 h at room temperature. Sections were incubated overnight at 4 °C with the following primary antibodies: CD8 (YTS169.4, 1:200, Abcam), MHC-I (BE0077, 1:250, Bio X Cell), and Granzyme B (QA18A28, 1:300, BioLegend). After incubation with fluorophore-conjugated secondary antibodies (Alexa Fluor 594 [red] for CD8, Alexa Fluor 488 [green] for MHC-I, and Alexa Fluor 555 [yellow] for Granzyme B) and DAPI, stained sections were observed using an APX100 All-in-One Fluorescence Microscope (Olympus, Tokyo, Japan).

### Cytokine and chemokine analyses

Supernatants secreted from CT26 cells treated with butyrate (0, 2 mM) for 24 h were subjected to multi-cytokine and chemokine assays using a mouse cytokine array kit (R&D Systems, Minneapolis, MN, USA) and ELISA for CXCL10 using Mouse IP-10 ELISA Kit (ab260067, Abcam), according to the manufacturers’ protocols.

### In vivo experiments

In a subcutaneous tumor model, CT26 cells (1 × 10^6^ cells) or HCT116 cells (1 × 10^6^ cells) were subcutaneously inoculated into the flanks of 6-week-old female BALB/c mice or BALB/c nude mice. Butyrate was administered orally via daily drinking water at a concentration of 1 mM. OBP-702 was intratumorally injected at a dose of 5 × 10^8^ PFUs 3 times a week and injected into the tumor on one side only in a bilateral subcutaneous tumor model. Oral administration of butyrate began 2 days prior to the first OBP-702 injection. The perpendicular diameter of each tumor was measured 3 times a week, and tumor volume was calculated using the following formula: tumor volume (mm^3^) = *a* × *b*^*2*^ × 0.5, where *a* represents the longest diameter, *b* represents the shortest diameter, and 0.5 is a constant used to calculate the volume of an ellipsoid.

To establish an orthotopic colon tumor model with liver metastases, CT26 cells stably expressing luciferase (CT26-Luc) (1 × 10^6^ cells) were inoculated into the submucosal layer of the ileocecum of BALB/c mice, followed by injection of CT26-Luc cells (5 × 10^5^ cells) into the portal vein. Mice were treated with butyrate and OBP-702 following the same schedule and method used in a subcutaneous tumor model. OBP-702 was injected only into the colon tumor under laparotomy. Tumor growth was monitored using an IVIS imaging system (Xenogen), and survival was assessed by Kaplan–Meier analysis.

The mice were housed in a specific pathogen-free environment in the Department of Animal Resources of Okayama University. All animal experimental protocols were approved by the Institutional Animal Care and Use Committee of Okayama University (Approval No. 2022818 for a subcutaneous tumor model and Approval No. OKU-2022769 for an orthotopic tumor model with liver metastases).

### Statistical analysis

All statistical analyses were performed using EZR (Saitama Medical Center, Jichi Medical University, Saitama, Japan), which is a graphical user interface for R (The R Foundation for Statistical Computing, Vienna, Austria). More precisely, it is a modified version of R commander that was designed to add frequently used biostatistical functions. Pearson’s chi-squared test or Fisher’s exact test was used for categorical variables, and the Mann–Whitney U test was used for continuous variables. A p value less than 0.05 was considered significant.

## Results

### Direct synergistic effects of butyrate and OBP-702

**Fig. 1:**
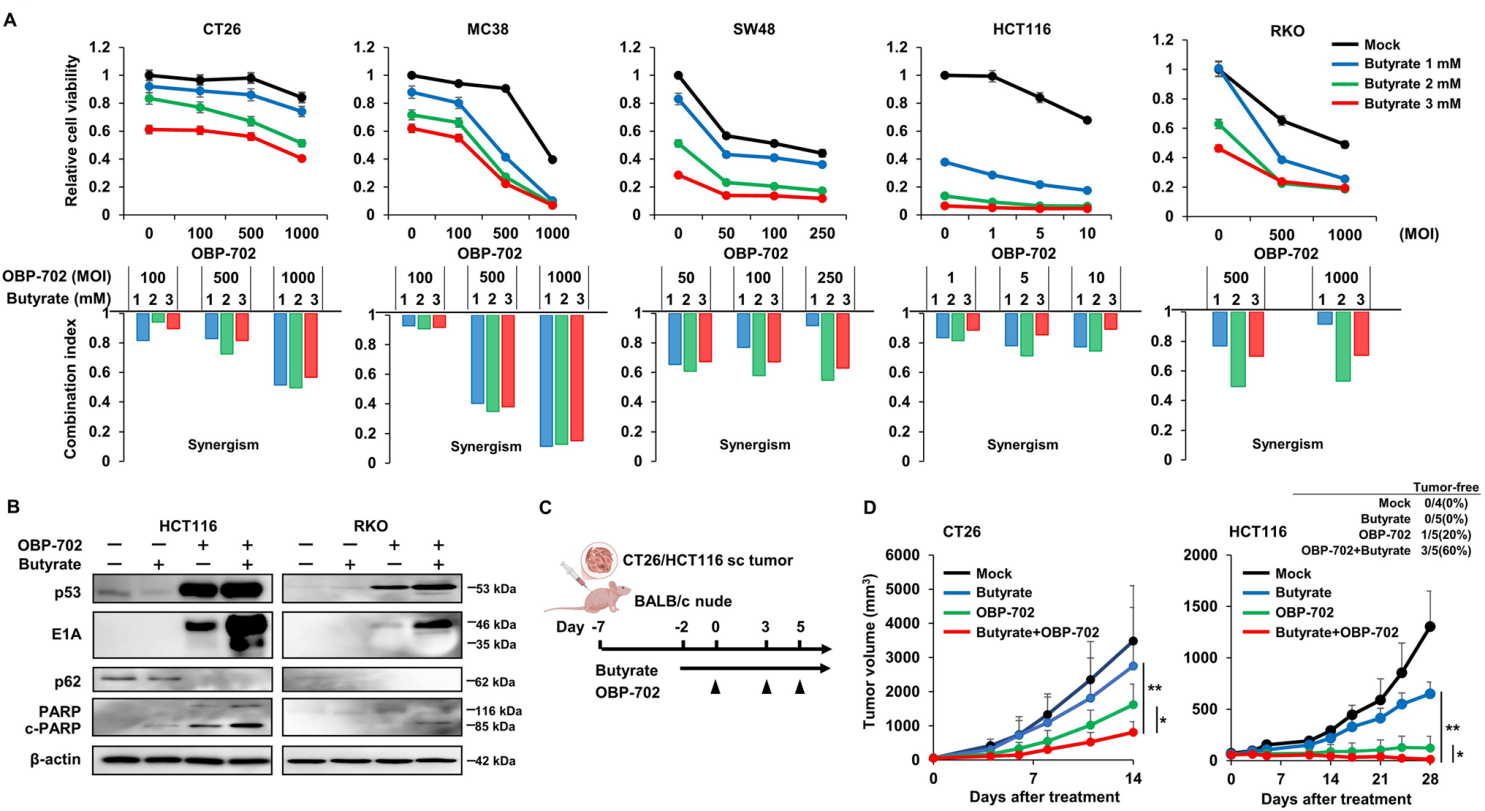
Direct synergistic effects of butyrate and OBP-702 **A** CT26, MC38, SW48, HCT116, and RKO cells were treated with OBP-702 at the indicated doses (MOI) and butyrate at concentrations of 0, 1, 2, and 3 mM, administered 2 h prior to OBP-702, and cell viability was assessed using an XTT assay 72 h after OBP-702 treatment (n = 3–5). The top panels show relative cell viability compared to mock-treated cells. Error bars indicated 95% confidence intervals. The bottom panels show combination index (CI) values calculated using CalcuSyn software. Synergy was defined as a CI < 1, and antagonism was defined as a CI > 1. **B** HCT116 and RKO cells were treated with butyrate (2 mM) and/or OBP-702 (5 MOI for HCT116 and 500 MOI for RKO) for 72 h, and the whole-cell lysates were analyzed by western blot for p53, E1A, p62, PARP, cleaved PARP (c-PARP), and β-actin. **C** Study protocol. Briefly, in a CT26 or HCT116 subcutaneous tumor model using immunodeficient BALB/c nude mice, tumors were treated with OBP-702 (5 × 10^8^ PFUs) intratumorally 3 times a week and/or butyrate (1 mM) orally, starting 2 days prior to the first OBP-702 injection. sc, subcutaneous. **D** Tumor volumes of CT26 (Mock group: n = 4; Butyrate, OBP-702, and Butyrate + OBP-702 group: n = 5) and HCT116 (Mock group; n = 4, Butyrate, OBP-702, and Butyrate + OBP-702 group: n = 5) were monitored until 14 days and 28 days after the first OBP-702 injection, respectively. *, *p* < 0.05. **, *p* < 0.01. The table shows the percentages of complete response for each treatment

When the cytotoxic effects of butyrate alone were first evaluated in vitro, butyrate demonstrated dose-dependent cytotoxicity on both colorectal cancer cell lines (CT26, MC38, SW48, HCT116) and a normal fibroblast cell line (MEF) (Fig. S2A), which was not caused by the acidic nature of butyrate (Fig. S2B, C). Similarly, although OBP-702 monotherapy also induced dose-dependent cytotoxicity in various human and murine colorectal cancer cell lines (CT26, MC38, SW48, HCT116, RKO), the combination of butyrate and OBP-702 exhibited synergistic cytotoxic effects across most concentrations, from low to high doses, in all these cell lines (Fig. 1A). This synergistic effect was attributed to the enhancement of autophagy and apoptosis, as shown by western blot analysis in which the combination of butyrate and OBP-702 led to downregulation of p62 and upregulation of c-PARP on HCT116 and RKO cells, although downregulation of p62 on RKO was not evident (Fig. 1B). When the direct combination effects of butyrate and OBP-702, independent of immune system mediation, were examined in vivo using immunodeficient BALB/c nude mice (Fig. 1C), the combination therapy of oral butyrate intake and OBP-702 intratumoral injection produced significantly stronger antitumor effects on CT26 and HCT116 subcutaneous tumors than each monotherapy, and, notably, in an HCT116 tumor model, 3 of 5 mice (60%) became tumor-free after this combination therapy, whereas 1 of 5 mice (20%) after OBP-702 monotherapy and 0 of 5 mice (0%) after butyrate monotherapy became tumor-free (Fig. 1D, Fig. S2D). These findings suggested that the combination therapy of butyrate and OBP-702 exhibited synergistic effects under immunodeficient conditions by enhancing autophagy and apoptosis.

## Enhancement of viral infectivity by butyrate

**Fig. 2:**
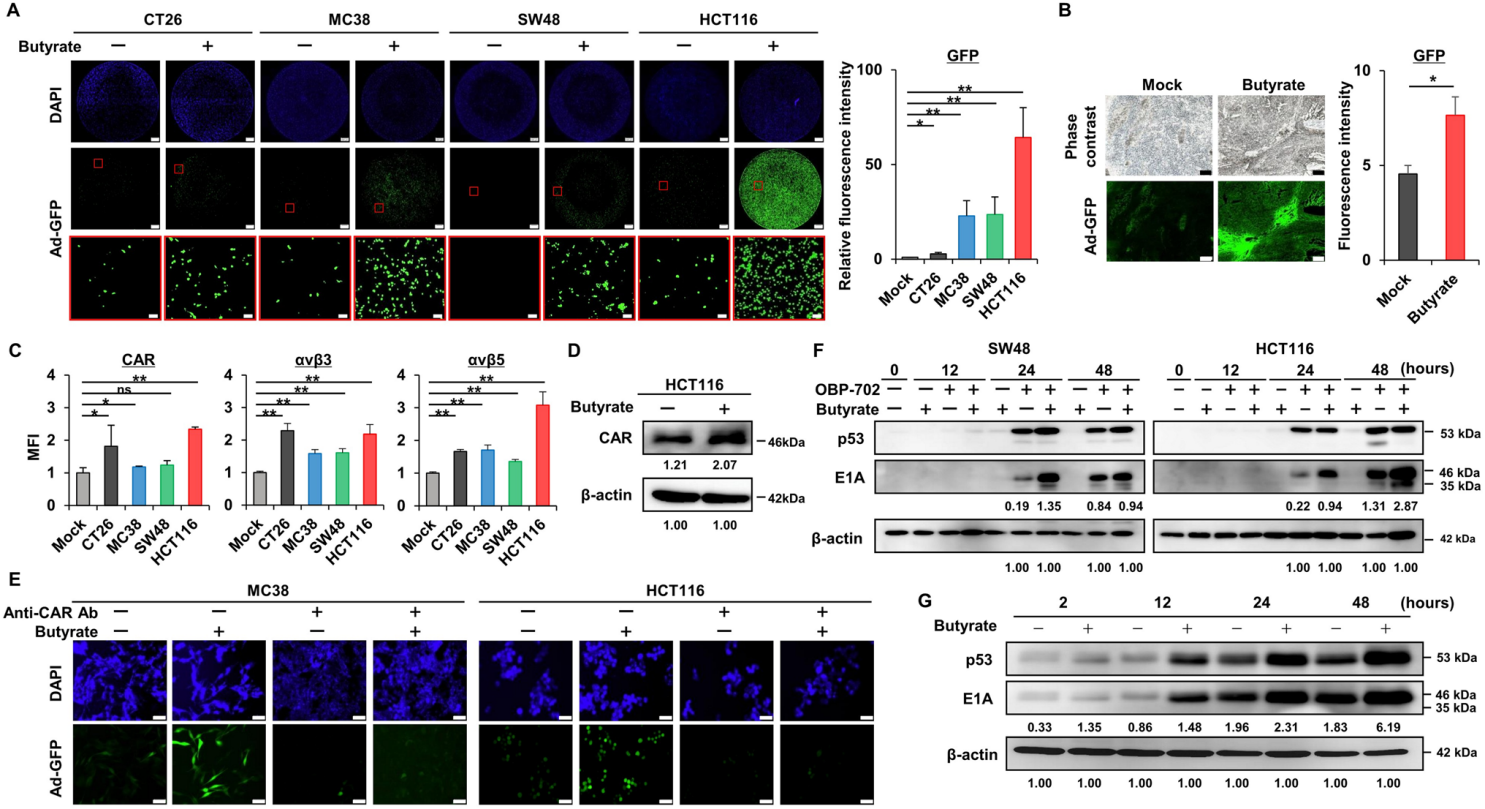
Enhancement of viral infectivity by butyrate **A** CT26, MC38, SW48, and HCT116 cells were treated with Ad-GFP (1000 MOI for CT26 and MC38, 100 MOI for SW48 and HCT116) with or without butyrate (2 mM) for 24 h, and were observed by fluorescence microscopy (n = 3–4). The lower panels show magnified views of the areas outlined by the red squares in the middle panels. Relative GFP intensity in butyrate-treated cells compared with mock-treated cells was measured in a randomly selected field in each well. Scale bar, 500 μm (upper and middle panels); 50 μm (lower panels). *, *p* < 0.05. **, *p* < 0.01. **B** HCT116 subcutaneous tumors were treated with Ad-GFP (5 × 10^7^ PFU) intratumoral injection for 2 days with or without butyrate (1 mM) oral administration, starting 2 days prior to Ad-GFP injection (n = 6). Frozen sections of harvested tumors with or without butyrate treatment were observed by fluorescence microscopy, and GFP intensity was measured in three randomly selected fields in each tumor. Scale bar, 200 μm. *, *p* < 0.05. **C** CT26, MC38, SW48, and HCT116 cells were treated with butyrate (2 mM) for 2 days, and analyzed with flow cytometry for CAR, αvβ3, and αvβ5 (n = 3). MFI, mean fluorescence intensity. **D** HCT116 subcutaneous tumors were treated with or without butyrate (1 mM) oral administration for 7 days, and whole-cell lysates of the harvested tumors were analyzed by western blot for CAR and β-actin. Relative intensity of CAR compared with β-actin is indicated below the CAR bands. **E** MC38 and HCT116 cells were treated with Ad-GFP (1000 MOI for MC38, 100 MOI for HCT116) with or without butyrate (2 mM) for 24 h. Anti-CAR antibody was administered 2 h prior to Ad-GFP infection. These cells were observed by fluorescence microscopy. Scale bar, 50 μm. **F** SW48 and HCT116 cells were treated with butyrate (2 mM) and/or OBP-702 (10 MOI) for 0, 12, 24, or 48 h, and the whole-cell lysates were analyzed by western blot for p53, E1A, and β-actin. Relative intensity of E1A compared with β-actin is indicated below the E1A bands. **G** HCT116 subcutaneous tumors were treated with OBP-702 (5 × 10^7^ PFU) intratumoral injection for 2, 12, 24, or 48 h with or without butyrate (1 mM) oral administration, starting 2 days prior to OBP-702 injection. Whole-cell lysates of the harvested tumors were analyzed by western blot for p53, E1A, and β-actin. Relative intensity of E1A compared with β-actin is indicated below the E1A bands

As a mechanism for the synergistic effects of butyrate and OBP-702, the effect of butyrate on viral infectivity was first examined using Ad-GFP, a replication-deficient adenovirus expressing GFP, on CT26, MC38, SW48, and HCT116 cells. Butyrate significantly enhanced viral infection in all these cell lines (Fig. 2A). Oral intake of butyrate also significantly increased viral uptake into tumors in an HCT116 subcutaneous tumor model (Fig. 2B). To understand the mechanism behind this increased infectivity, the effects of butyrate on expressions of coxsackievirus and adenovirus receptor (CAR), αvβ3, and αvβ5, which are molecules involved in adenoviral infection, were examined. Butyrate significantly upregulated CAR, αvβ3, and αvβ5 expressions on CT26, MC38, SW48, and HCT116 cells (Fig. 2C). Upregulation of CAR was also observed in an in vivo model using HCT116 subcutaneous tumors (Fig. 2D). The importance of CAR in enhancing adenoviral infectivity by butyrate was demonstrated by a CAR inhibition assay in MC38 and HCT116 cells, showing that pre-treatment with an anti-CAR antibody abrogated the enhancement of Ad-GFP infection by butyrate (Fig. 2E). In contrast, butyrate did not affect the replication efficiency of adenovirus in SW48 and HCT116 cells (Fig. S3A). When butyrate was combined with OBP-702 in vitro, butyrate significantly enhanced adenoviral E1A expression on SW48 and HCT116 cells as early as 2 h after OBP-702 treatment, before replication began, indicating increased infectivity (Fig. S3B). This enhancement of adenoviral E1A expression by butyrate was maintained at 24 and 48 h after sufficient replication on SW48 and HCT116 cells, although p53 expression was not significantly enhanced in HCT116 cells (Fig. 2F). In an HCT116 subcutaneous tumor model, 2-day pretreatment with butyrate enhanced p53 and E1A expressions at 2, 12, 24, and 48 h after OBP-702 treatment (Fig. 2G). These findings suggested that the direct synergistic effects of butyrate and OBP-702 were mediated by the enhancement of the initial infectivity of OBP-702 via CAR and integrins, rather than by its effect on viral replication efficiency.

## Immune modulation by butyrate

**Fig. 3:**
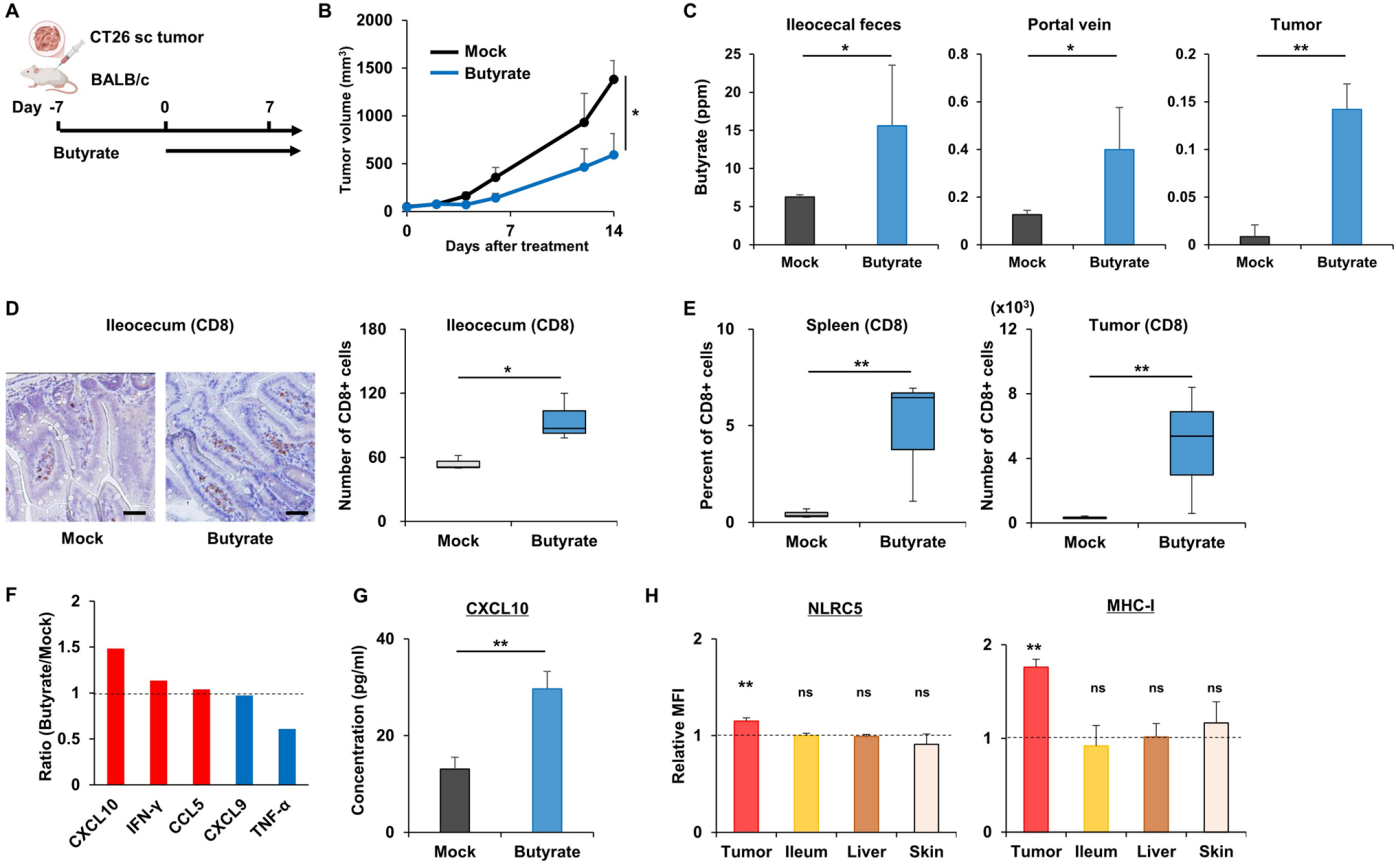
Immune modulation by butyrate **A** Study protocol. Briefly, in a CT26 subcutaneous tumor model using immunocompetent BALB/c mice, tumors were treated with mock or butyrate (1 mM) orally. sc, subcutaneous. **B** Tumor volumes of CT26 were monitored until 14 days after treatment initiation (n = 3). *, *p* < 0.05. **C** Feces of the ileocecum, blood from the portal vein, and the tumor were harvested from BALB/c mice bearing CT26 subcutaneous tumors 14 days after the initiation of oral butyrate intake (n = 5). Butyrate concentrations in these samples were analyzed by gas chromatography mass spectrometry. **D** Paraffin-embedded sections of the ileocecum harvested 14 days after the initiation of oral butyrate intake were immunostained with CD8 and observed by microscopy (n = 3). The number of CD8-positive cells was measured in a randomly selected field in each tumor. Scale bar, 50 μm. *, *p* < 0.05. **E** The spleen and the tumor harvested 14 days after the initiation of oral butyrate intake were analyzed by flow cytometry for CD8 (n = 3). **, *p* < 0.01. **F** Conditioned media from CT26 cells treated with mock or butyrate (2 mM) for 24 h in vitro were analyzed by a multi-cytokine and chemokine assay (n = 2). Relative concentrations of CXCL10, IFN-γ, CCL5, CXCL9, and TNF-α after butyrate treatment compared to mock treatment are shown. **G** Conditioned media from CT26 cells treated with mock or butyrate (2 mM) for 48 h in vitro were analyzed by ELISA for CXCL10 (n = 3). **, *p* < 0.01. **H** The tumor, ileum, liver, and skin harvested 14 days after the initiation of oral butyrate intake were analyzed by flow cytometry for NLRC5 and MHC-I (n = 3). **, *p* < 0.01. ns, not significant. MFI, mean fluorescence intensity

Prior to experiments examining the combination effects of butyrate and OBP-702 under immunocompetent conditions, the effect of butyrate alone on the immune system was first assessed using immunocompetent BALB/c mice (Fig. 3A). Oral butyrate intake itself significantly suppressed the growth of CT26 subcutaneous tumors with minimal adverse events, as indicated by the absence of notable body weight loss, reduced activity, poor coat condition, diarrhea, or mortality at any butyrate dose (Fig. 3B, Fig. S4A). The butyrate concentration was significantly increased not only in the ileocecum, but also in the portal vein and the tumor tissue 14 days after oral intake (Fig. 3C). Then, CD8 + T cells were significantly increased in the lumen of the ileocecum, spleen, and tumor tissue (Fig. 3D,E). Tregs, which are immunosuppressive and mainly suppress the function of effector T cells, were also significantly increased in the ileocecum and tumor tissue, but not in the spleen (Fig. S4B,C). In in vitro experiments, butyrate modulated multiple cytokines and chemokines, including CXCL10, IFN-γ, and CCL5 (Fig. 3F, Fig. S4D). Notably, butyrate significantly increased CXCL10 secretion by CT26 cells, a key chemokine that activates antitumor immunity (Fig. 3G), and upregulated MHC-I expression by colorectal cancer cell lines (CT26, MC38, SW48, HCT116), but not a normal fibroblast (MEF) line (Fig. S4E). In a CT26 subcutaneous tumor model, butyrate significantly increased NLRC5 expression, a key regulator of MHC-I, selectively in the tumor tissue, but not in the other organs such as the ileum, liver, and skin, which led to the upregulation of MHC-I in the tumor tissue alone, with no significant effect on the ileum, liver, and skin (Fig. 3H). These findings suggested that butyrate itself had modest antitumor activity and systemic immune modulatory effects.

## Synergistic effects of butyrate and OBP-702 via antitumor immunity

**Fig. 4:**
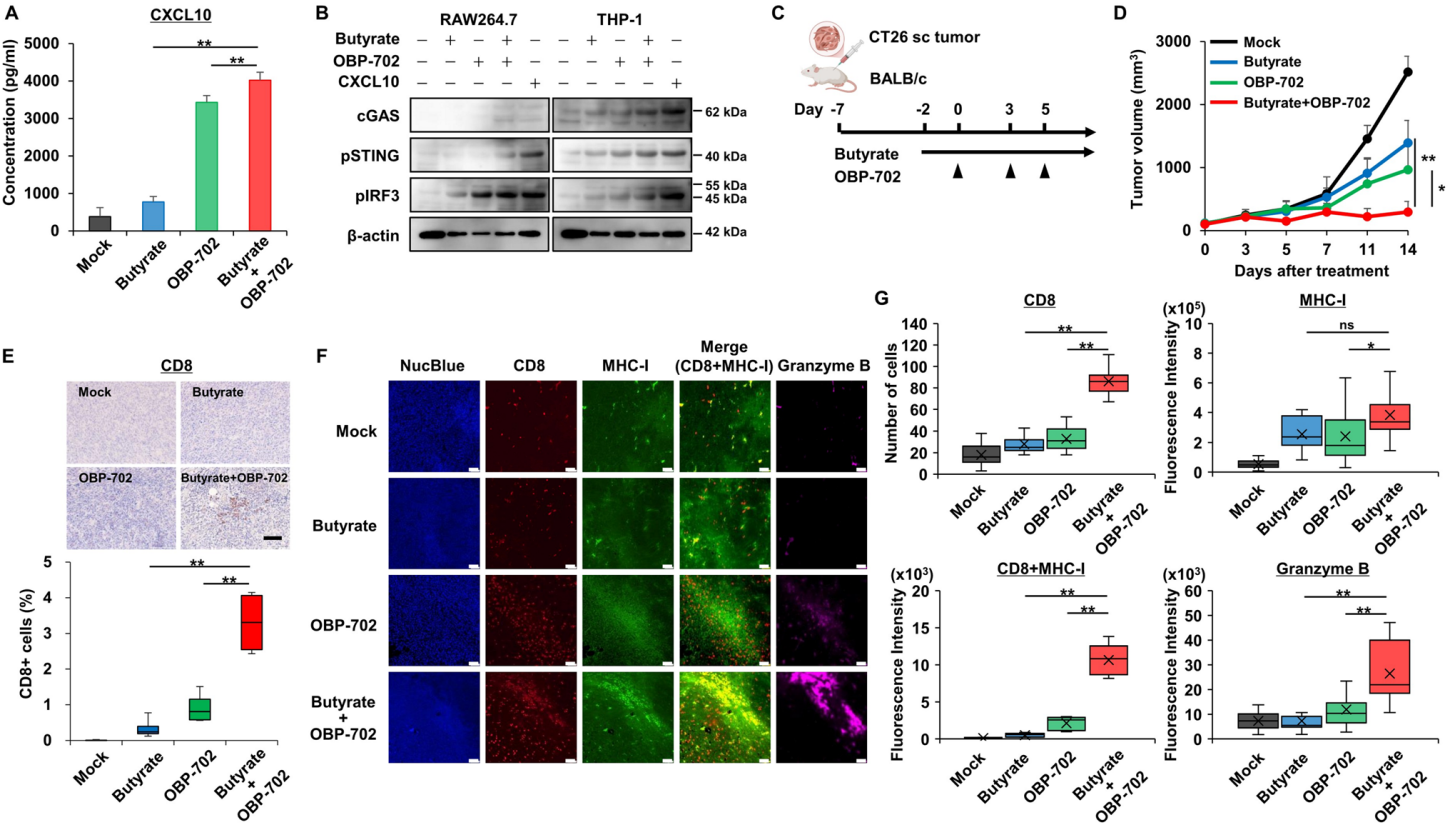
Synergistic effects of butyrate and OBP-702 via antitumor immunity **A **Conditioned media from CT26 cells treated with butyrate (2 mM) and/or OBP-702 (1000 MOI) for 48 h were analyzed by ELISA for CXCL10 (n = 5). **, *p* < 0.01. **B** RAW264.7 and THP-1 cells were co-cultured for 24 h with conditioned media from CT26 cells treated with butyrate (2 mM) and/or OBP-702 (1000 MOI) for 48 h, and the whole-cell lysates were analyzed by western blot for cGAS, pSTING, pIRF3, and β-actin. CXCL10 recombinant was used as a stimulator of the cGAS-STING-IRF3 pathway. **C** Study protocol. Briefly, in a CT26 subcutaneous tumor model using immunocompetent BALB/c mice, tumors were treated with OBP-702 (5 × 10^7^ PFUs) intratumorally 3 times a week and/or butyrate (1 mM) orally, starting 2 days prior to the first OBP-702 injection. sc, subcutaneous. **D** Tumor volumes of CT26 were monitored until 14 days after the first OBP-702 injection (n = 4). *, *p* < 0.05. **, *p* < 0.01. **E** Paraffin-embedded sections of the tumors harvested 14 days after the first OBP-702 injection were immunostained with CD8 and observed by microscopy (n = 4). The percent of CD8-positive cells in area was measured in three randomly selected fields. Scale bar, 100 μm. **, *p* < 0.01. **F** Representative images of multiplex fluorescence immunostaining for Nucleus (Blue), CD8 (red), MHC-I (green), Merged CD8 and MHC-I (yellow), and Granzyme B (magenta) of the tumors. Scale bar, 50 μm. **G** Quantification of CD8, MHC-I, Merged CD8 and MHC-I, and Granzyme B based on the images taken in F (n = 5). *, *p* < 0.05. **, *p* < 0.01

When the combination effects of butyrate and OBP-702 on antitumor immunity were first examined in vitro, this combination significantly increased CXCL10 secretion by CT26 cells compared with butyrate or OBP-702 alone (Fig. 4A). When the effect of this combination on the cGAS-STING-IRF3 pathway, an important pathway of MHC-I regulation, was examined using RAW264.7 and THP-1 cells co-cultured with conditioned media from CT26 cells treated with butyrate and/or OBP-702, the combination of butyrate and OBP-702 strongly stimulated the cGAS-STING-IRF3 pathway in both RAW264.7 and THP-1 cells, similar to the effect of recombinant CXCL10 (Fig. 4B). Next, when the antitumor effects of this combination were examined in a CT26 subcutaneous tumor model using immunocompetent BALB/c mice (Fig. 4C), the combination therapy of oral butyrate intake and OBP-702 intratumoral injection significantly suppressed tumor growth compared with each monotherapy (Fig. 4D, Fig. S5A), whereas adverse events such as body weight loss, reduced activity, poor coat condition, diarrhea, or mortality were minimal with both the combination therapy and each monotherapy (Fig. S5B). In the analyses of antitumor immunity, the combination therapy of butyrate and OBP-702 significantly recruited CD8 + T cells (Fig. 4E) and increased MHC-I expression in the tumor tissues harvested 14 days after the first OBP-702 injection, compared with each monotherapy (Fig. S5C). Further analysis by immunofluorescence staining of the same tumor tissues showed that increased CD8 + T cells by the combination therapy were strongly merged with increased MHC-I expression, and were further merged with granzyme B, a serine protease of cytotoxic T cells that induces apoptosis in target cells (Fig. 4F,G). These findings suggested that the combination therapy of butyrate and OBP-702 produced strong antitumor activity through an antitumor immune response via CD8 + T cell activation and MHC-I upregulation mediated by the cGAS-STING-IRF3 pathway.

## Abscopal effects by combination butyrate and OBP-702 therapy

**Fig. 5:**
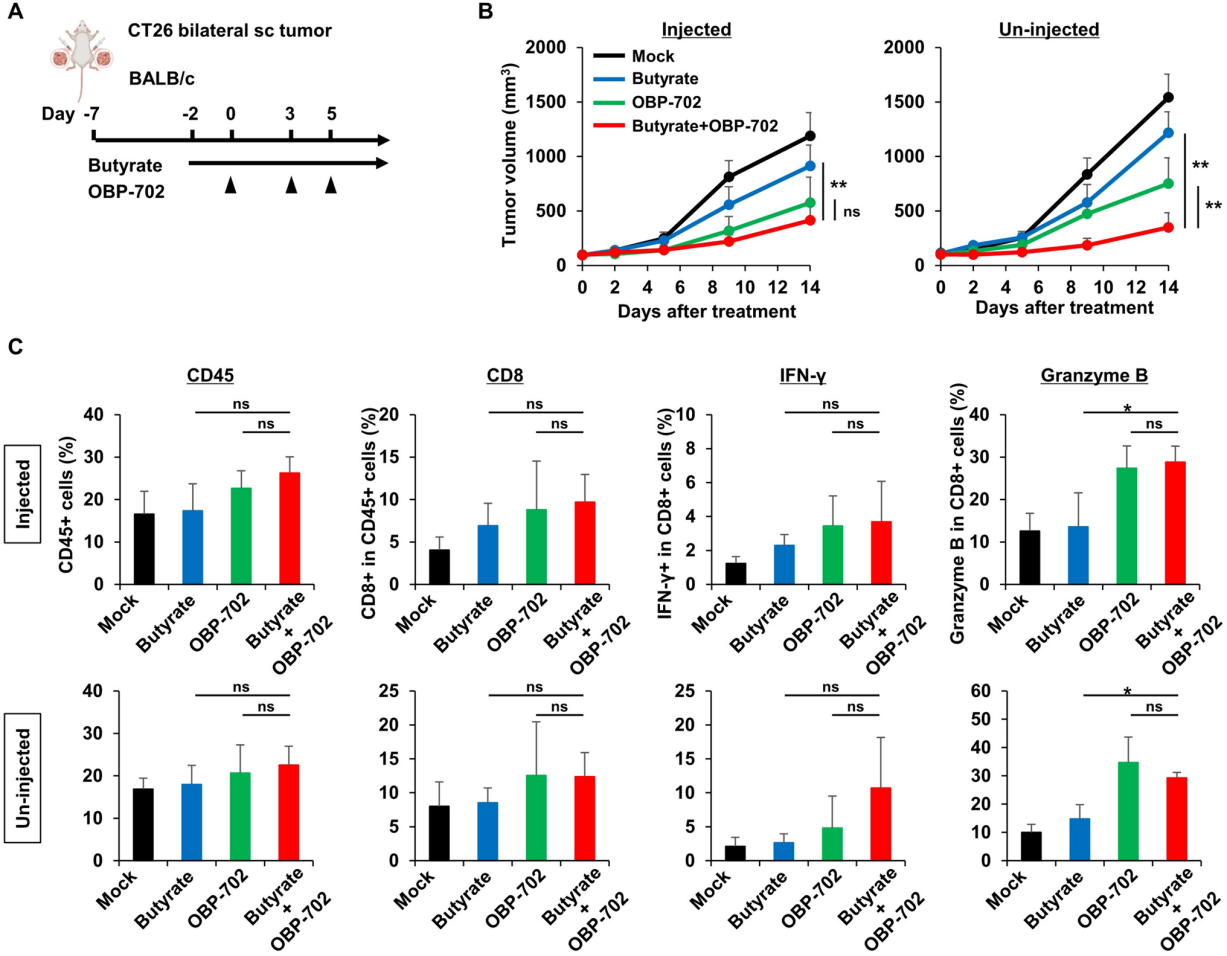
Abscopal effects in a bilateral subcutaneous tumor model **A** Study protocol. Briefly, in a CT26 bilateral subcutaneous tumor model using immunocompetent BALB/c mice, mice were treated with OBP-702 (5 × 10^7^ PFUs) intratumorally 3 times a week and/or butyrate (1 mM) orally, starting 2 days prior to the first OBP-702 injection. OBP-702 was injected only into the tumor on one side. sc, subcutaneous. **B** Tumor volumes of CT26 on both sides were monitored until 14 days after the first OBP-702 injection (n = 4–5). ns, not significant. **, *p* < 0.01. **C** The tumor tissues on both sides harvested 14 days after the first OBP-702 injection were analyzed by flow cytometry for CD45, CD8, IFN-γ, and Granzyme B (n = 4). ns, not significant. *, *p* < 0.05

Finally, the abscopal effects on metastatic tumors, which were not directly injected with OBP-702, were examined in a CT26 bilateral subcutaneous tumor model and a CT26-Luc orthotopic colon tumor model with liver metastases using immunocompetent BALB/c mice. First, in a CT26 bilateral subcutaneous tumor model (Fig. 5A), the combination therapy of oral butyrate intake and OBP-702 intratumoral injection on one side significantly suppressed tumor growth on both the OBP-702 injected- and un-injected sides, although no significant difference was observed on the injected side compared with OBP-702 monotherapy (Fig. 5B, Fig. S6). In the analysis of antitumor immunity using the tumor tissues of both sides harvested 14 days after the first OBP-702 injection, the combination therapy of butyrate and OBP-702 tended to increase CD8 + T cells, CD8 + IFN-γ + T cells, and Granzyme B expression in the tumor tissues on both sides. However, no significant differences were observed, except in Granzyme B expression compared with butyrate monotherapy (Fig. 5C).

**Fig. 6:**
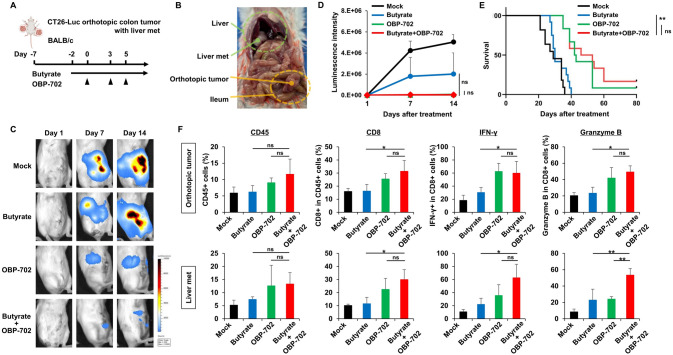
Abscopal effects in an orthotopic colon tumor model with liver metastases **A** Study protocol. Briefly, in a CT26-Luc orthotopic colon tumor model with liver metastases using immunocompetent BALB-c mice, mice were treated with OBP-702 (5 × 10^7^ PFUs) intratumorally 3 times a week and/or butyrate (1 mM) orally, starting 2 days prior to the first OBP-702 injection. OBP-702 was injected only into the colon tumor under laparotomy. met, metastasis. **B** Macroscopic image of the orthotopic colon tumor and the liver metastasis under laparotomy. met, metastasis. **C** Representative IVIS images of each treatment group taken 1, 7, and 14 days after the first OBP-702 injection. **D** Luciferase activity of each treatment group was quantified and plotted (n = 5–9). ns, not significant. **E** The Kaplan–Meier survival curves of each treatment group (n = 11–12). ns, not significant. **, *p* < 0.01. **F** The orthotopic tumors and the liver metastases harvested 14 days after the first OBP-702 injection were analyzed by flow cytometry for CD45, CD8, IFN-γ, and Granzyme B (n = 4). met, metastasis. ns, not significant. *, *p* < 0.05. **, *p* < 0.01

Next, in a CT26-Luc orthotopic colon tumor model with liver metastases (Fig. 6A,B), the combination therapy of oral butyrate intake and OBP-702 injection into the colon tumor strongly suppressed tumor growth, as demonstrated by IVIS imaging, although no significant differences were observed compared with each monotherapy (Fig. 6C,D). On survival analysis, the combination therapy significantly prolonged the survival period of mice compared with butyrate monotherapy, although no significant difference was observed compared with OBP-702 monotherapy (Fig. 6E). In the analysis of antitumor immunity using the tumor tissues of the orthotopic and metastatic sites harvested 14 days after the first OBP-702 injection, the combination therapy of butyrate and OBP-702 significantly increased CD8 + T cells, CD8 + IFN-γ + T cells, and Granzyme B expression in the orthotopic tumors and the liver metastases compared with butyrate monotherapy. In addition, the combination therapy significantly increased Granzyme B in the liver metastases compared with OBP-702 monotherapy (Fig. 6F). These findings suggested that the combination therapy of butyrate and OBP-702 had the potential to produce the abscopal effect on metastatic sites via systemic antitumor immune activation.

## Discussion

The gut microbiota plays a critical role in maintaining immune homeostasis, and butyrate or butyrate-producing bacteria are a key element in this function. Butyrate has been reported to exert immunomodulatory effects and activate antitumor immunity by increasing MHC-I levels in tumor cells and inducing CXCL10 secretion, leading to CD8 + T cell recruitment, which were also confirmed in the present study. Regarding its effect on Tregs, butyrate has been shown to recruit Tregs in the gut and mitigate inflammation in inflammatory bowel disease. In the present study, oral butyrate intake increased Foxp3 + Tregs in the tumor tissue, as well as in the gut, which may act as an inhibitory factor for antitumor immunity. Nevertheless, the present data suggest that the overall antitumor effect of butyrate was preserved, likely because CD8 + T cell activation and MHC-I upregulation outweighed the suppressive influence of Tregs, consistent with previous reports indicating that Treg induction can function as a counter-regulatory mechanism [24, 25]. Given these immunomodulatory properties, butyrate has attracted great attention as a potential combination partner for several treatment modalities, including immune checkpoint blockade.

Oncolytic virotherapy is a promising treatment strategy that exerts antitumor efficacy by novel mechanisms distinct from existing standard therapies such as chemotherapy, radiotherapy, and immunotherapy. Oncolytic adenoviruses including OBP-301 and OBP-702 that we have been developing have characteristics that induce tumor specific cytotoxicity following CAR- and integrin-dependent infection and hTERT promoter-driven replication. In addition, they activate the systemic antitumor immune response via immunogenic cell death, with abscopal effects on the metastatic site expected even after local treatment. Whereas monotherapy with oncolytic adenoviruses can produce decent antitumor efficacy against various types of cancers, we have demonstrated that combinations with other treatment strategies such as chemotherapy, radiotherapy, immune checkpoint inhibitors, and others provide more profound therapeutic benefits. One such combination involves HDAC inhibitors, and we previously reported that an HDAC inhibitor enhanced adenovirus infectivity by upregulating CAR, leading to improved therapeutic potential of oncolytic adenoviruses [26]. In the present study, the combination effects with butyrate, which has been reported to function as an HDAC inhibitor, were investigated, and the synergistic mechanisms of butyrate and OBP-702 were elucidated from the two aspects, direct effects of butyrate on OBP-702 therapeutic potential and indirect effects of butyrate mediated through antitumor immunity.

As for direct effects, butyrate enhanced the cytotoxic activity of OBP-702 by increasing its infectivity in tumor cells via CAR and integrins, a mechanism likely attributed to its function as an HDAC inhibitor, as previously reported [27]. This effect was observed on a variety of colorectal cancer cell lines in vitro and in vivo, and an inhibition assay of Ad-GFP infection using an anti-CAR antibody further supported this finding. Butyrate has been reported to enhance cellular infection not only with adenovirus, but also with other viruses, including influenza virus, reovirus, and HIV-1, whereas there is also a report indicating that a butyrate releaser exerted a protective action against SARS-CoV-2 infection [28]. Regarding its effect on virus replication, butyrate seemed to have a minimal effect on the replication of OBP-702, although a study reported that butyrate promoted virus infection and replication via repression of IFN-stimulated genes [29]. In addition, the replication efficiency of OBP-702 is driven by the human hTERT promoter, whose transcriptional activity is known to be substantially lower in murine cells than in human cells [30, 31]. Therefore, viral replication is likely less efficient in murine colorectal cancer cell lines such as CT26 and MC38 than in human colorectal cancer cells, which may partly explain the requirement for higher MOIs in murine models. The present study focused on CRC because oral butyrate intake is presumed to have a direct effect on CRC, but given that the concentration of butyrate in subcutaneous tumors increased significantly following oral intake, similar effects may be expected in other types of tumors such as pancreatic cancer.

As for indirect effects, butyrate activated the antitumor immune response by promoting CD8 + T cell activation and upregulating MHC-I expression via the cGAS-STING-IRF3 pathway, a mechanism also associated with its function as an HDAC inhibitor. Notably, MHC-I expression was selectively upregulated in the tumor tissues following oral butyrate intake, leading to enhanced antitumor activity via the immune system with minimal adverse events. OBP-702 local injection itself has the potential to activate systemic antitumor immunity and induce abscopal effects on the metastatic site, and the addition of oral butyrate intake is expected to augment this effect. However, in the present study, some inconsistencies regarding this additional effect of butyrate on the abscopal effect were observed. In a bilateral subcutaneous tumor model, significant additional antitumor effects (OBP-702 vs Butyrate + OBP-702) were observed on the OBP-702 un-injected side, whereas no significant differences were observed in the analyses of antitumor immunity such as CD8, IFN-γ, and Granzyme B. In an orthotopic tumor model, significant additional effects were observed only in the analysis of Granzyme B in the liver metastases, which may be attributed to OBP-702 monotherapy being too potent to allow for additional effects of butyrate, as suggested by the IVIS imaging results. From this perspective, immunologically cold tumors such as pancreatic cancer may represent a more suitable target for this treatment strategy. Nevertheless, it should be acknowledged that, in both the bilateral subcutaneous and orthotopic models, although the combination therapy produced clear suppression of tumor growth and a survival benefit, the immune marker changes were limited. This may be partly explained by the strong baseline potency of OBP-702 monotherapy, which may have masked the additional immunological effects of butyrate. In addition, tumor microenvironment heterogeneity and the relatively small sample sizes may have further contributed to the lack of statistical significance. These limitations suggest that further studies with larger cohorts and optimized viral dosing will be required to more precisely delineate the immunological contributions of the combination therapy.

Although butyrate was used in the present study, probiotics producing butyrate such as *Clostridium butyricum* will be more suitable for clinical application. In fact, *Clostridium butyricum* therapy has been reported to enhance the efficacy of immune checkpoint inhibitors in patients with lung cancer [32]. Though the efficacy of combination therapy with *Clostridium butyricum* and an oncolytic virus has not yet been reported, this strategy holds promise and could be a highly attractive approach. To further advance this concept, future studies should explore the potential of gut microbiota modulation, particularly the use of butyrate-producing probiotics, as a novel adjunct to oncolytic virotherapy. Given that gut microbiota composition is highly dynamic and affected by diet, lifestyle, and medication, personalized approaches leveraging microbiome profiling could be instrumental in identifying patients who may benefit the most from such combination therapies. At the same time, potential risks should also be taken into consideration. These include interindividual variability in microbiome composition that can influence butyrate production, challenges in controlling the effective in vivo dose of butyrate, and possible gastrointestinal side effects such as bloating or diarrhea. Recent reports have emphasized that responses to butyrate or synbiotic supplementation vary depending on host and microbial contexts, underscoring the need for careful patient selection and dose optimization in future clinical applications (33, 34).

**Fig. 7:**
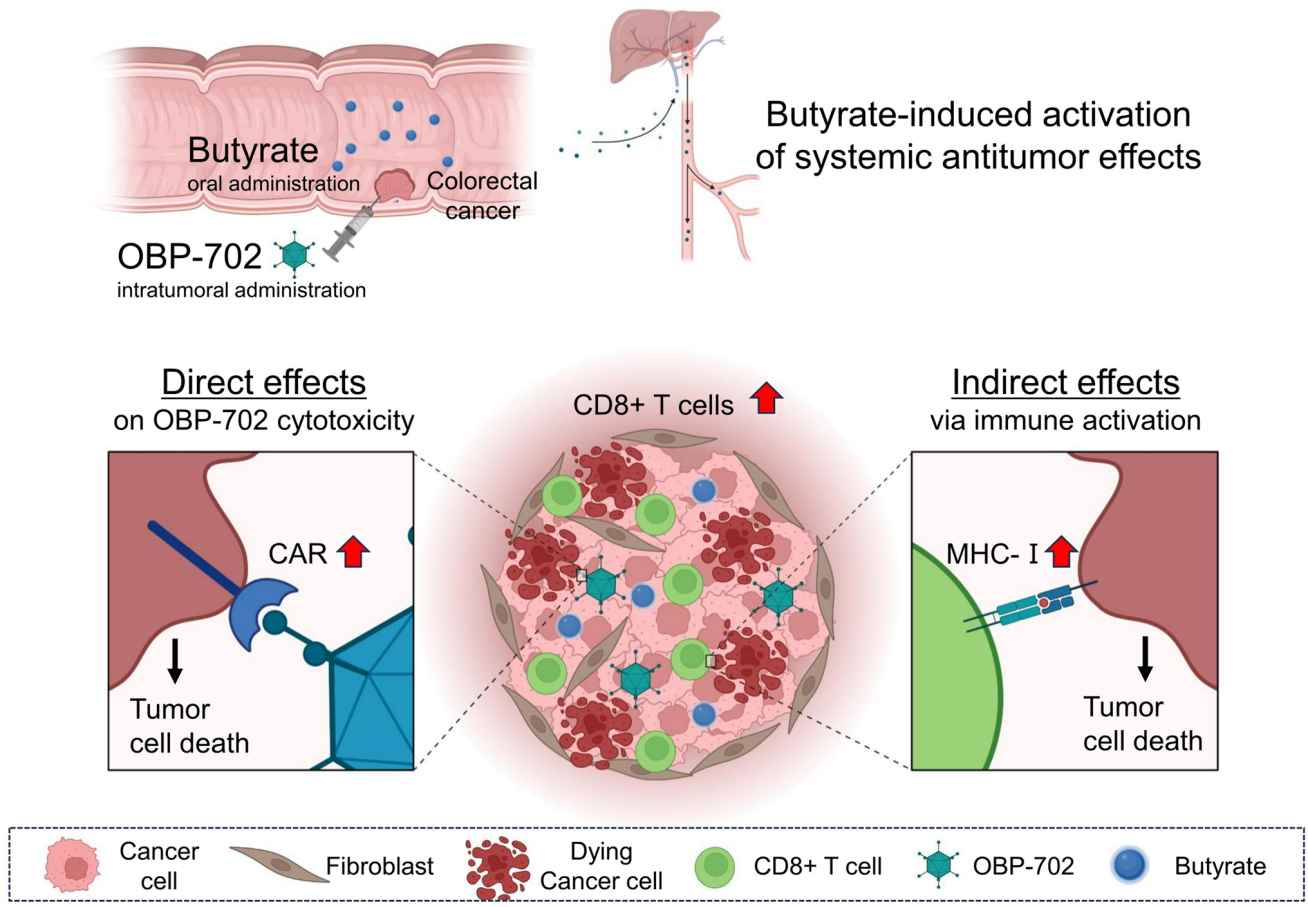
Schematic illustration

In conclusion, this study highlights the potential of combining butyrate with oncolytic adenovirus therapy as a means to enhance tumor-specific immune responses and improve treatment outcomes (Fig. 7). Though further research is warranted, particularly in immunologically cold tumors such as pancreatic cancer, the present findings provide a strong rationale for integrating gut microbiota-targeted interventions into cancer immunotherapy. The emerging interplay among gut microbiota, viral oncolysis, and immune modulation represents a promising frontier in the development of next-generation cancer therapies, with the potential to significantly impact clinical practice in the near future.

## Supplementary Information

Below is the link to the electronic supplementary material.

Supplementary file1 (PDF 573 KB)

## Data Availability

The data generated in this study are available upon request from the corresponding author.
