# Supplementary material for: Gut microbial metabolite butyrate boosts p53-expressing telomerase-specific oncolytic adenovirus efficacy by enhancing infectivity and activating MHC-I/cGAS-STING
Source: Cancer Immunol Immunother. 2025 Dec 18;75(1):10. doi: 10.1007/s00262-025-04252-4 (PMC12715064; doi:10.1007/s00262-025-04252-4)
Supplement: Supplementary file 1 — Supplementary file1 (PDF 573 KB) [file 262_2025_4252_MOESM1_ESM.pdf]

Supplementary Information

Gut microbial metabolite butyrate boosts p53-expressing telomerase-specific oncolytic adenovirus efficacy by enhancing infectivity and activating MHC-I/cGAS-STING

Masaki Sakamoto[1], Shinji Kuroda[1*], Tetsuya Katayama[1], Yu Mikane[1], Shunya Hanzawa[1], Daisuke Kadowaki[1], Yusuke Yoshida[1], Yuki Hamada[1], Ryoma Sugimoto[1], Chiaki Yagi[1], Masashi Hashimoto[1], Nobuhiko Kanaya[1], Yoshihiko Kakiuchi[1], Satoru Kikuchi[1], Kunitoshi Shigeyasu[1], Hiroshi Tazawa[1,2], Shunsuke Kagawa[1], Yasuo Urata[3], Toshiyoshi Fujiwara[1]

[1] Department of Gastroenterological Surgery, Faculty of Medicine, Dentistry and Pharmaceutical Sciences, Okayama University, Okayama, Japan.

[2] Center for Innovative Clinical Medicine, Okayama University Hospital, Okayama, Japan.

[3] Oncolys BioPharma, Inc., Tokyo, Japan.

Supplementary Fig. S1 Gene structure of OBP-702

Supplementary Fig. S2 Supporting data for Fig. 1

Supplementary Fig. S3 Supporting data for Fig. 2

Supplementary Fig. S4 Supporting data for Fig. 3

Supplementary Fig. S5 Supporting data for Fig. 4

Supplementary Fig. S6 Supporting data for Fig. 5

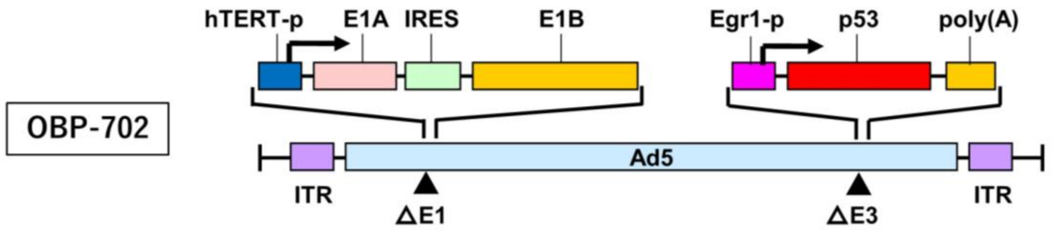

Supplementary Fig. S1 Gene structure of OBP-702

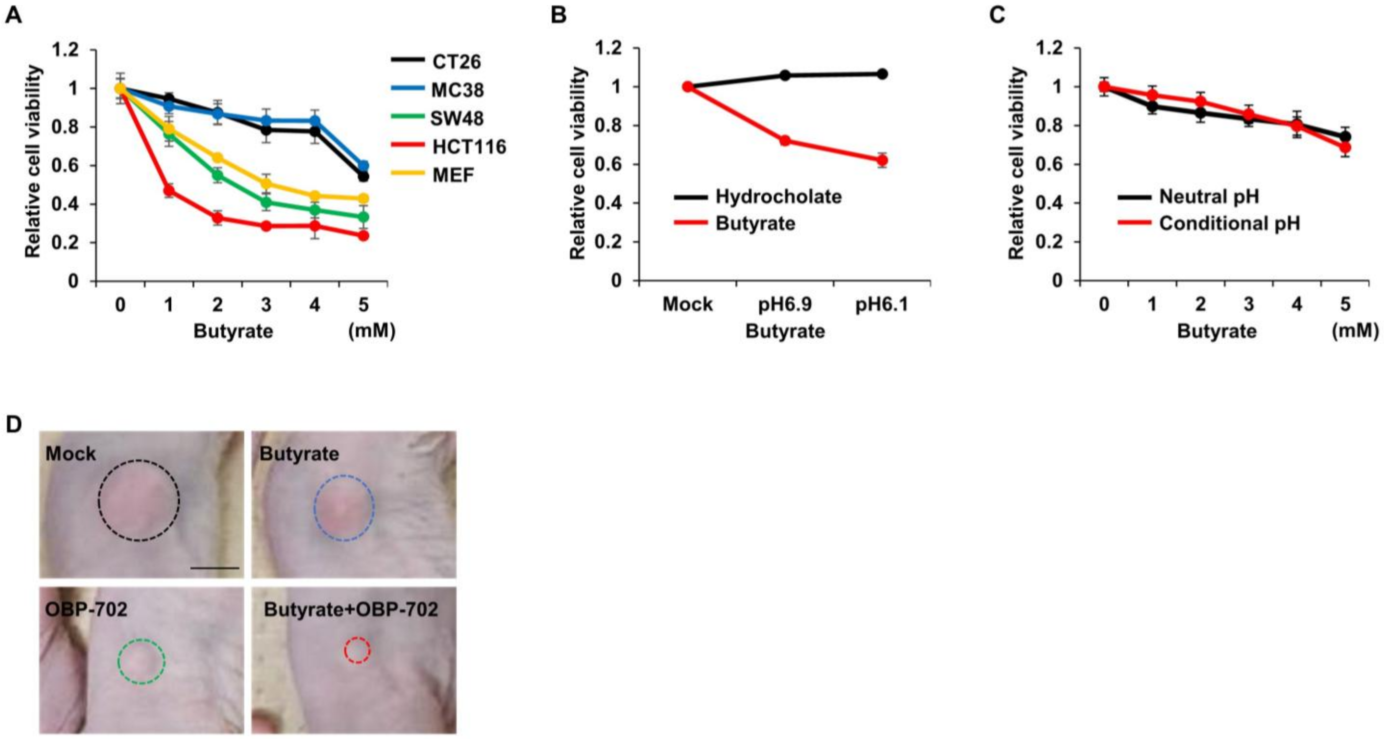

Supplementary Fig. S2 Supporting data for Figure 1

A CT26, MC38, SW48, HCT116, and MEF cells were treated with butyrate at the indicated concentrations, and cell viability was assessed using an XTT assay 72 hours after treatment (n=5). Relative cell viability compared with mock-treated cells. Error bars indicate 95% confidence intervals. **B** CT26 cells were treated with hydrochloric acid or butyrate at matched pH levels (pH 7.4, 6.9, 6.1) for 48 hours, and cell viability was assessed using an XTT assay (n=5). Relative cell viability compared with mock-treated cells is plotted. Error bars indicate 95% confidence intervals. **C** CT26 cells were treated with butyrate at various concentrations (0, 1, 2, 3, 4, and 5 mM) either without pH correction or with pH adjusted to 7.4 using NaOH for 48 hours, and cell viability was assessed using an XTT assay (n=3). Relative cell viability compared with mock-treated cells is plotted. Error bars indicate 95% confidence intervals. **D** Representative macroscopic images of HCT116 subcutaneous tumors 28 days after treatments of mock, butyrate, OBP-702, or the combination. Scale bar, 1 cm.

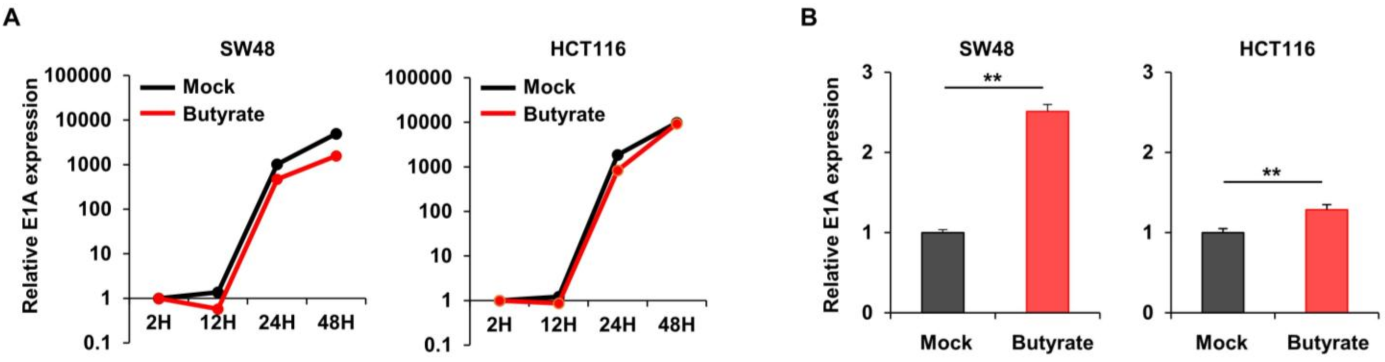

Supplementary Fig. S3 Supporting data for Figure 2

A SW48 and HCT116 cells were treated with mock or butyrate (2 mM) 2 hours prior to OBP-702 (10 MOI) infection, and were harvested 2, 12, 24, and 48 hours after OBP-702 infection (n=3). The extracted DNA was analyzed by qRT-PCR analysis for adenoviral E1A gene levels. E1A copy numbers are described as fold change relative to 2 hours. **B** SW48 and HCT116 cells were treated with mock or butyrate (2 mM) 2 hours prior to OBP-702 (10 MOI) infection, and were harvested 2 hours after OBP-702 infection (n=3-5). The extracted DNA was analyzed by qRT-PCR analysis for adenoviral E1A gene levels. E1A copy numbers are described as fold change relative to mock treatment. **, $p < 0.01$.

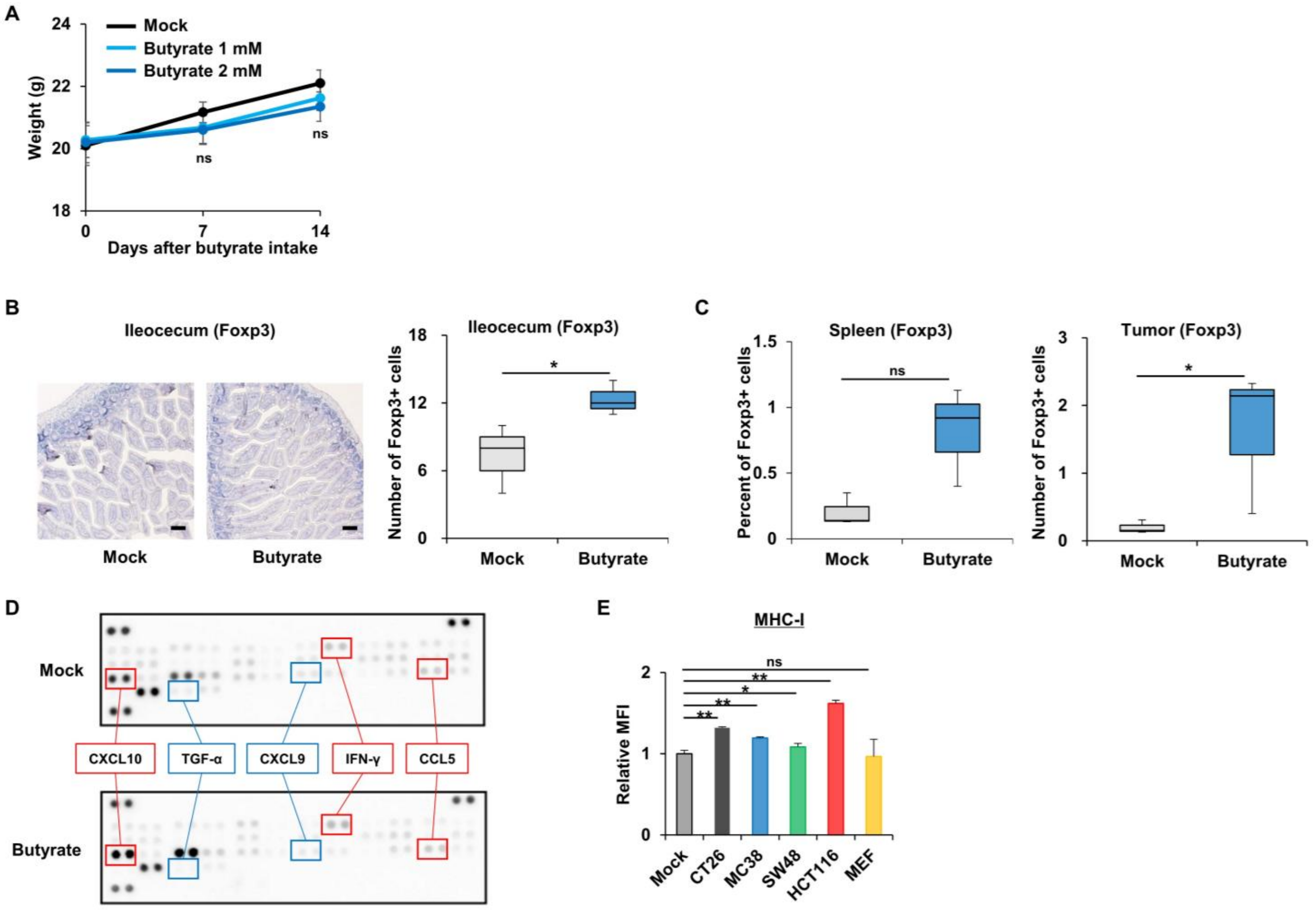

Supplementary Fig. S4 Supporting data for Figure 3

A BALB/c mice were treated with mock, 1 mM butyrate, or 2 mM butyrate orally, and body weight was measured for toxicity assessment 0, 7, and 14 days after treatment initiation (n=3-4). ns, not significant. **B** In a CT26 subcutaneous tumor model using BALB/c mice, tumors were treated with mock or butyrate (1 mM) orally, and the ileocecum was harvested 14 days after the initiation of oral butyrate intake (n=3). Paraffin-embedded sections were immunostained with Foxp3 and observed by microscopy. The number of Foxp3-positive cells was measured in a randomly selected field in each tumor. Scale bar, 50 µm. *, $p < 0.05$. **C** The spleen and the tumor harvested 14 days after the initiation of oral butyrate intake were analyzed by flow cytometry for Foxp3 (n=3). ns, not significant. *, $p < 0.05$. **D** Membranes of multi-cytokine and chemokine assay. The spots of CXCL10, IFN-γ, CCL5, CXCL9, and TNF-α are displayed. **E** CT26, MC38, SW48, HCT116, and MEF cells were treated with mock or butyrate (2 mM) for 48 hours, and were analyzed by flow cytometry for MHC-I (n=3). ns, not significant. *, $p < 0.05$. **, $p < 0.01$. MFI, mean fluorescence intensity.

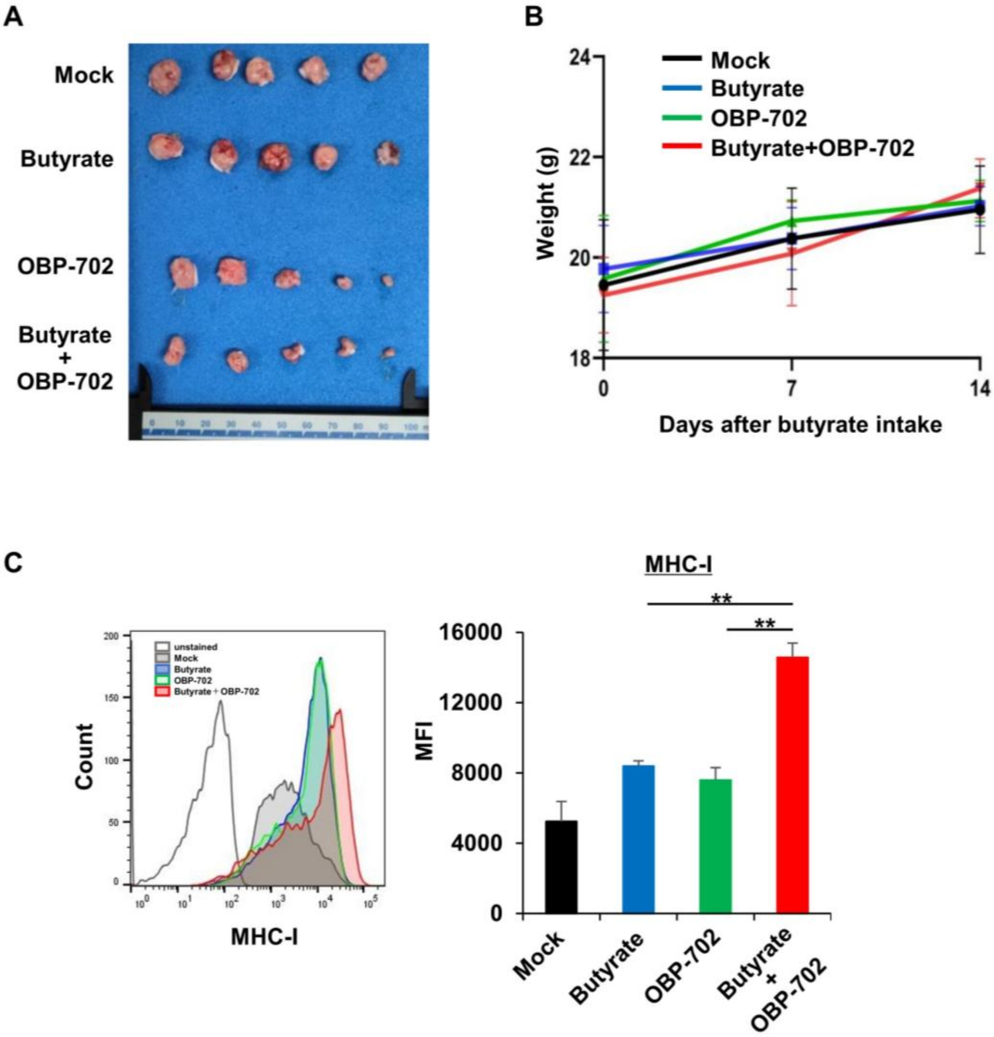

Supplementary Fig. S5 Supporting data for Figure 4

A In a CT26 subcutaneous tumor model using BALB/c mice, tumors were treated with OBP-702 (5×10^7 PFUs) intratumorally 3 times a week and/or butyrate (1 mM) orally, starting 2 days prior to the first OBP-702 injection. Macroscopic image of tumors harvested 14 days after each treatment are shown. **B** The body weight of mice in each group was monitored for 14 days after treatment. **C** The tumor tissues harvested 14 days after each treatment were analyzed by flow cytometry for MHC-I (n=3). **, $p < 0.01$. MFI, mean fluorescence intensity.

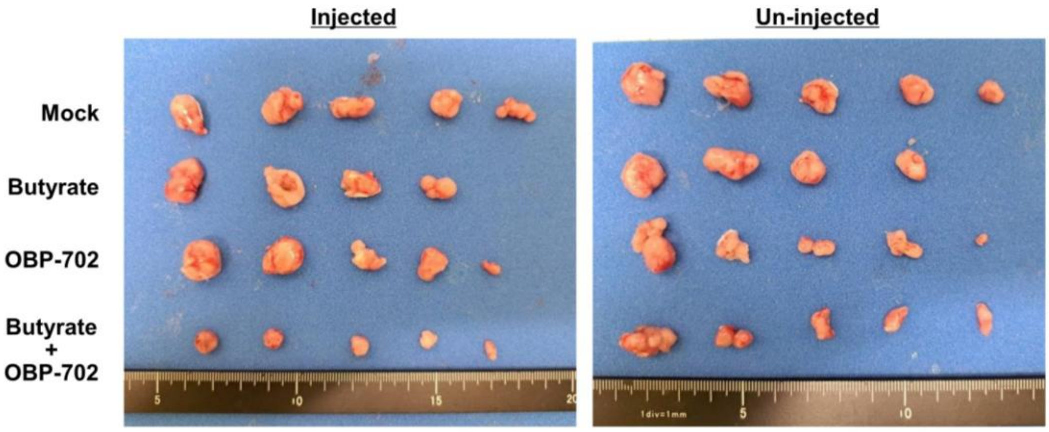

Supplementary Fig. S6 Supporting data for Figure 5

In a CT26 bilateral subcutaneous tumor model using immunocompetent BALB/c mice, mice were treated with OBP-702 (5×10^7 PFUs) intratumorally 3 times a week and/or butyrate (1 mM) orally, starting 2 days prior to the first OBP-702 injection. OBP-702 was injected only into the tumor on one side. Macroscopic image of tumors harvested 14 days after each treatment are shown.
